# Supplementary material for: Evaluation of a training program for rheumatic heart disease screening integrated into the public health system in Uganda
Source: PLoS One. 2026 Mar 30;21(3):e0344012. doi: 10.1371/journal.pone.0344012 (PMC13035113; doi:10.1371/journal.pone.0344012)
Supplement: S1 Fig — (DOCX) [file pone.0344012.s001.docx]

| **Goal** | **Timing** | **Approach** | **Tools/Resources** | **Minimum Standards** |
| --- | --- | --- | --- | --- |
| Initial Training | No more than 30 days prior to in-person training | Obtain background knowledge relating to ultrasound and RHD | 10-Part Tablet-based Moodle Modules | ≥80% on all modules *(can repeat modules if needed)* |
|  | Week 1 Training | In-person Lectures | ADUNU training team, trainee health facility | Attend at least 90% of in-person training |
|  | Week 1 Training | Hands on scanning with trainer | Ugandan Heart Institute technical team, using handheld ultrasound device | Completion of ten supervised scans |
|  | Week 1 Training | Case Review – Pathology | 50 Case Moodle Module | Completion of Module |
| Assessment | End of Week 1 | Scanning Practical Exam (2 observed scans, scanning technique, machine use, patient positioning, image quality) | ADUNU training team, trainee health facility | Trainer deems trainee competent (able to obtain images)  *(Personalized remediation if needed)* |
|  | End of Week 1 | Case Review – Pathology | 25 Case Moodle Assessment | 100% correctly identified as screen positive or screen negative *(repeat 50 case Moodle module and try again as needed)* |
| Initial Certification | If pass training assessment | Eligible if they attended ≥80 of training sessions and completed 10 independent scans during the training week |  | Successful completion of practical exam and Moodle assessment |
| Audit and feedback | Weeks 2-14+ | Preform scans without supervision | Scans performed on handheld ultrasound devices (Lumify) and uploaded to remote cloud database (Tricefy) and reviewed by team of six cardiologists in Uganda and US. Biweekly reviews with results communicated back to trainees by the ADUNU training team. ADUNU also provides ongoing education and intermittent group review of missed cases. | Complete ≥20 scans/month |
| Re-training | Variable, ≥3 months after conditional certification | Booster sessions individualized to the needs of each trainee that is not yet meeting final certification criteria. | ADUNU training team at trainee’s home health center or the district hospital | Attend single day booster session |
| Final  Certification | Variable | Based on cardiologist interpretation of studies | Cardiologist review of completed studies | At least 12 weeks, at least 100 studies, and maintains an accuracy of >90% for 2 consecutive weeks |
