# Supplementary material for: Evaluation of a training program for rheumatic heart disease screening integrated into the public health system in Uganda
Source: PLoS One. 2026 Mar 30;21(3):e0344012. doi: 10.1371/journal.pone.0344012 (PMC13035113; doi:10.1371/journal.pone.0344012)
Supplement: S2 Fig — (DOCX) [file pone.0344012.s002.docx]

| **Phase** | **Materials** | **Requirement** |
| --- | --- | --- |
| Phase 1: Initial Health Center Training (1 week) | | |
| 1a: Pre-Education | ED app-based learning module | 1. Complete module within 30 days of starting Phase 1b. |
| 1b: Initial Training  (1 week) | Lecture-based didactic | 1. Attend didactic session |
|  | Hands-on scanning under direct supervision | 1. Once provider has completed 15 scans (including two independent scans) they are eligible for the test. 2. If fails, must complete 15 more scans (including two independent scans) and retest on Friday. 3. If fails again, send to remediation pathway. |
|  | Pattern Recognition Practice | 1. 5 rapid-fire quiz modules (one per day) comprising 25 echo cases. Must obtain 100% on each module to pass. Can retake as many times as needed. This ensures appropriate pattern recognition of RHD on screening echocardiogram. |
| Conditional Certification |  | Satisfactorily complete all three requirements.   1. Didactic requirement 2. Scanning requirement 3. Module requirement   If fails any of three by Friday, send to remediation pathway. |
| Phase 2: Independent Reinforcement of Skills (6  weeks) | | |
| 2a: Independent Scanning Period | Facility Logs | 1. Goal to independently perform 100 scans by 6 weeks following conditional certification. 2. ADUNU team to upload screening log data into REDCap weekly. 3. Feedback on number of scans reported to providers weekly. |
|  | Site Visit Audits of Independently Performed Scans | 1. Two “site” visits by district team. Providers are encouraged to write down challenging cases they would like to discuss further. |
|  | Weekly Pattern Recognition Practice | 1. 5 rapid-fire quiz modules (one per week, accounting for one week missed for travel, other duties, etc) comprising 25 echo cases on same content as above (1b). Must get >90% (can retake the weekly module if score < 90%) |
| Final Certification |  | Complete 100 scans, pass (>90%) all 5 weekly quiz/modules. If not meeting these criteria can extend the independent scanning period up to 8 weeks following conditional certification. If has not passed by 8 weeks, send to remediation pathway. |
| Phase 3: Maintenance of Certification | | |
| 3a: Monitoring of Referrals | Log at facility, log at referral center | 1. An RHD focus person will perform quarterly audits of each provider to quantify total # scans, total # referrals, proportion of accurate referrals (true positives). 2. These metrics will be compiled for each provider, then compared to the total group. If provider falls outside the 80%ile, the RHD focal person will support that individual with tailored content. |
| 3b: Continued Education | Pattern Recognition Practice | 1. Provider to complete 1 rapid fire quiz/module of 25 questions each quarter. Must obtain > 90% score. Can re-take 3 times. If fails after four attempts, then send to remediation pathway. |
| Phase 4: Remediation / Off-Cycle Training | | |
| Remediation and off-cycle training will be offered concurrently to minimize the burden on the RHD focal person and the training team from Ugandan Heart Institute. If there is a new trainee, they will complete the full cycle above, but the location of their training might be moved from the health center to the centralized referral center (e.g., district hospital). Remediation and off-cycle training will be offered a minimum of twice yearly, with the health care worker repeating the full course. | | |
