# Supplementary material for: Evaluation of a training program for rheumatic heart disease screening integrated into the public health system in Uganda
Source: PLoS One. 2026 Mar 30;21(3):e0344012. doi: 10.1371/journal.pone.0344012 (PMC13035113; doi:10.1371/journal.pone.0344012)
Supplement: Attachment — Submitted filename: RHD UGANDA REVEIW.docx [file pone.0344012.s003.docx]

This is an important manuscript on rheumatic heart disease (RHD) screening training in Uganda. Its an ambitious and timely work that addresses a major public health gap in Sub-Saharan Africa. The study is well-structured, with clear methodology and valuable findings. I believe this paper deserves publication, provided the following issues are carefully addressed:

**Major Points for Improvement**

1. **Tables and Data Presentation**

Several tables are overloaded with details, which may reduce readability. Please simplify by separating *inputs/resources* from *outcomes* or moving highly technical cost details to supplementary material.

Ensure that percentages and denominators are always clear (e.g., specify the “n” for conditional vs final certification groups in tables).

2. **Results and Statistics**

The results are descriptive, which is acceptable, but the statistical presentation can be strengthened. Confidence intervals should be added for sensitivity/specificity estimates and for major proportions (e.g., final certification rates).

Consider logistic regression or stratified analysis to identify predictors of successful certification and sustained practice. This would add depth and highlight determinants of program success.

3. **Discussion**
   - The discussion is somewhat repetitive in places. Please sharpen the focus by directly contrasting your findings with other large-scale RHD screening experiences (e.g., Fiji, Brazil PROVAR, etc.).
   - Expand on the implications of the low sensitivity (59%), and propose concrete strategies for improving early detection (e.g., booster training, AI-assisted screening, embedding RHD into standard nursing curricula).
   - Please include *regional context*. There are now reports emerging from Horn of Africa, which provide valuable complementary evidence. We recommend citing:
     - Abdi IA, Karataş M, Öcal L, Ahmed SA, Hassan MS, Atilla K, Mohomud MF. *Pattern of rheumatic heart disease among patients attending at a tertiary care hospital in Somalia: first report from Somalia.* **Am J Cardiovasc Dis.** 2023 Oct 15;13(5):345.
     - Abdi IA, Karataş M, Öcal L, Elmi Abdi A, Farah Yusuf Mohamud M. *Retrospective analysis of left ventricular thrombus among heart failure patients with reduced ejection fraction at a single tertiary care hospital in Somalia.* **Open Access Emerg Med.** 2022;14:591–7.
   - Including these will help frame Uganda’s experience as part of a broader East African movement toward locally generated data and scalable solutions.
4. **Conclusion**
   - The conclusion should be more concise and action-oriented. Currently, it repeats parts of the discussion. Instead, emphasize three key take-home messages:
     1. RHD screening training is feasible in public health systems.
     2. Sensitivity remains suboptimal and requires iterative training/innovation.
     3. The model is scalable and should inform regional/global policies.

Despite these areas for improvement, I want to emphasize that this is a very strong and much-needed paper. With sharper presentation of the data, strengthened analysis, and improved discussion, this manuscript will make an important contribution to the global RHD literature. If the above revisions are approached carefully, I am confident the paper will be accepted for publication.
